# Management of extracranial carotid artery pseudoaneurysms

**DOI:** 10.1016/j.jvscit.2025.102021

**Published:** 2025-10-24

**Authors:** Yuliia Mota, Ihor Kobza, Taras Kobza, Rostyslav Zhuk, Yuriy Orel, Dmytro Beshley, Andriy Piliukh

**Affiliations:** a Department of Surgery No. 2, Danylo Halytsky Lviv National Medical University, Lviv, Ukraine; b Department of Vascular Surgery and Transplantology, Lviv Regional Clinical Hospital, Lviv, Ukraine; c Department of Endovascular, Ukrainian-Polish Heart Center Lviv, Lviv, Ukraine

**Keywords:** Carotid artery, Complications, Long-term results, Management, Pseudoaneurysm

## Abstract

**Objective:**

Extracranial carotid artery pseudoaneurysm (ECAPA) is a rare vascular pathology most commonly associated with trauma, infection, spontaneous dissection, iatrogenic injury, or prior surgery. Management of ECAPAs still remains uncertain, without specific recommendations included in the most recent guidelines. The aim of the study was to review the experience of a single center with ECAPAs during a 20-year period.

**Methods:**

For the period from December 2003 to February 2024, 14 patients with ECAPAs were prospectively included and retrospectively reviewed. Clinical signs, aneurysm profiles, and treatment outcomes were recorded. Patients underwent open surgery or endovascular intervention.

**Results:**

The underlying etiology of ECAPAs was infection in five patients (35.7%), trauma in four patients (28.6%), dissection in four patients (28.6%), and giant cell arteritis in one patient (7.1%). All patients were symptomatic, presenting with neck mass, swallowing disorders, transient ischemic attack, stroke, rupture, or postoperative wound infection. Surgical interventions included resection of the pseudoaneurysm with primary anastomosis in three patients (21.4%), carotid artery reimplantation in two patients (14.3%), carotid artery ligation in three patients (21.4%), extra-anatomic autogenous subclavian-carotid bypass in two patients (14.3%), prosthetic graft interposition in two patients (14.3%), and endovascular treatment in two patients (14.3%). Perioperative complications included: ischemic stroke in three patients (21.4%), transient cranial nerve dysfunction in two patients (14.3%), thrombosis of arterial reconstruction in two patients (14.3%), and wound infection in two patients (14.3%). The postoperative mortality rate was 7.1%. Long-term outcomes were assessed in 13 patients. Median follow-up was 21 months (range, 1-222 months). During follow-up, nine patients (69.2%) experienced no new neurological events, whereas one patient (7.7%) suffered a stroke. Seven patients (53.8%) showed no carotid stenosis or occlusion. Two patients (15.4%) were lost to follow-up, and one patient (7.7%) died due to cancer progression.

**Conclusions:**

ECAPA is a rare but potentially life-threatening condition that requires timely surgical intervention. The findings of this study suggest that reconstructive surgery can be an effective treatment, often resulting in favorable long-term outcomes. Further studies with larger cohorts and longer follow-up are needed to optimize management strategies and compare surgical vs endovascular approaches.

Extracranial carotid artery pseudoaneurysm (ECAPA) is a rare vascular pathology most commonly associated with trauma, infection, spontaneous dissection, iatrogenic injury, or previous surgery. Symptomatic patients typically present with a pulsating neck mass, swallowing disorders, or cranial nerve dysfunction, which are associated with aneurysm growth, distal embolization, and even rupture.^1^, ^2^, ^3^, ^4^

Management of ECAPAs still remains uncertain, as current guidelines provide no specific recommendations. Open surgery is considered the gold standard due to better long-term results and is the preferred approach in patients with mycotic pseudoaneurysms (PAs), mass effect, or active bleeding. Endovascular repair is alternative option, particularly in patients with distal PAs, previous neck surgery, or a history of radiation therapy.^4^, ^5^, ^6^ Nevertheless, optimal treatment strategy remains controversial and requires further investigation. The aim of the study was to review the experience of a single center with ECAPAs during a 20-year period.

## Methods

### Study design and patient selection

**Fig 1:**
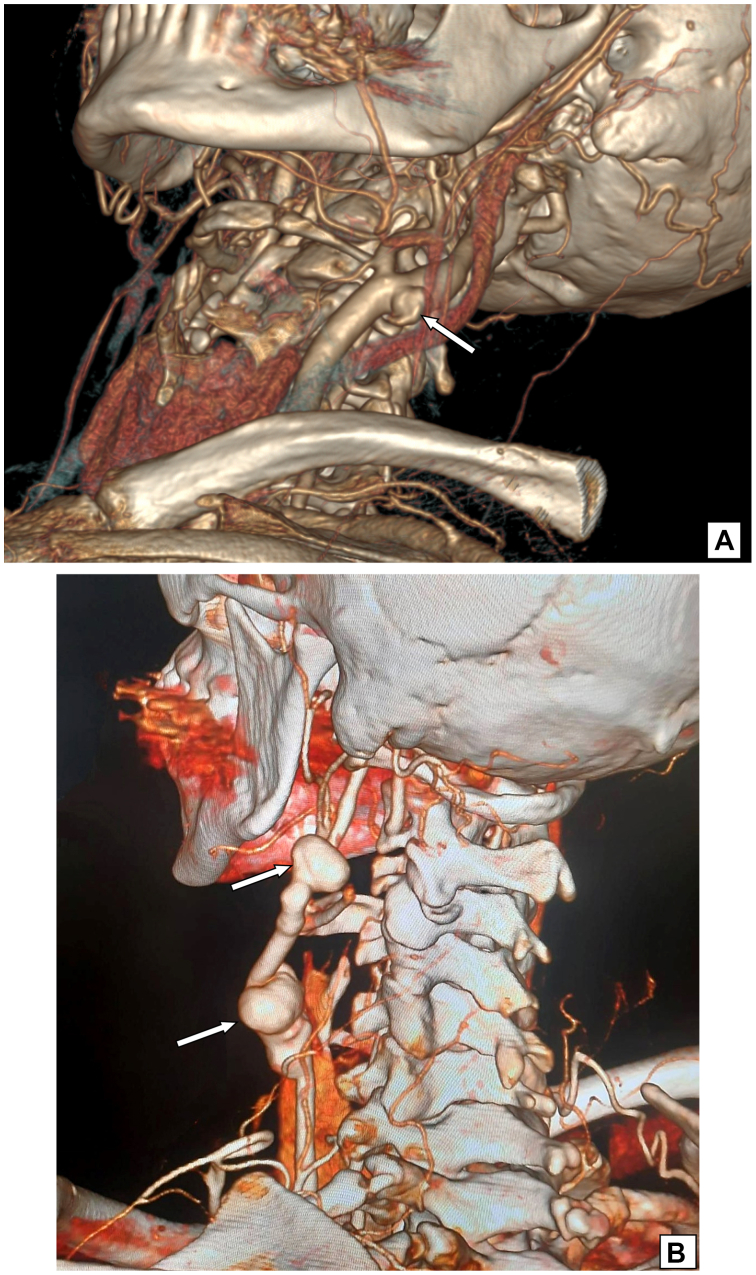
Computed tomography angiography of head and neck (three-dimensional reconstruction of neck vessels). **(A)** Pseudoaneurysm (PA) of left internal carotid artery due to giant cell arteritis; **(B)** PAs of the carotid vein graft anastomoses.

All patients treated for ECAPAs in the Vascular Surgery Department of Lviv Regional Clinical Hospital and Ukrainian-Polish Heart Center Lviv, from December 2003 to February 2024, were prospectively included and retrospectively analyzed. Both infectious (mycotic) and noninfectious PAs were included, regardless of etiology (trauma, dissection, infection, vasculitis, iatrogenic injury, or prior surgery). Clinical signs, aneurysm profile, and treatment outcomes were recorded. The diagnosis was based on duplex ultrasound followed by computed tomography angiography or magnetic resonance angiography (Fig 1).

### Anesthesia and operative strategy

Patients underwent open surgery or endovascular intervention. Local anesthesia was used in five patients (35.7%) to enable real-time neurological monitoring. General anesthesia was administered in the other nine cases (64.3%), mainly due to arterial rupture or critical patient condition requiring airway and circulatory control. Open surgical procedures included resection of PA with either primary anastomosis, carotid artery reimplantation, graft interposition, bypass grafting, or carotid artery ligation. The choice of surgical technique was based on aneurysm location, size, vessel condition, and presence of infection.

### Endovascular technique

Endovascular treatment was performed in two patients with distally located PAs of the internal carotid artery (ICA), which were anatomically inaccessible for open surgical repair. Procedures were carried out under local or general anesthesia via the right common femoral artery using a standard transfemoral approach. Following selective carotid angiography, covered stent grafts—Gore Viabahn (W. L. Gore & Associates) and Abbott Graftmaster (Abbott Vascular)—were deployed under fluoroscopic guidance to exclude the PA from circulation and maintain distal ICA patency.

### Outcomes and follow-up

Early results (<30 days) were evaluated in terms of mortality, perioperative stroke or transient ischemic attack, cranial nerve injuries, thrombosis of arterial reconstruction, and wound complications. After intervention patients were followed at 1, 3, and 6 months and then annually by clinical examination and duplex ultrasonography. Follow-up data were obtained from patients’ medical records.

### Statistical analysis

Descriptive statistics were used for analysis. Continuous variables were presented as mean ± standard deviation or median with range, and categorical variables as absolute numbers and percentages. Due to the small cohort size, no inferential statistical testing was performed. Data analysis was carried out using StatSoft STATISTICA version 12.

### Ethical approval

The study protocol was approved by the institutional ethics committee. Data was collected following ethical approval. All procedures conformed to the principles of the Declaration of Helsinki.

## Results

Fourteen patients were included in the study: 13 adults (mean age, 49.7 ± 13.0 years) and one pediatric patient aged 1 year and 7 months. Twelve patients (85.7%) were male, and two patients (14.3%) were female. PAs involved the ICA in nine patients (64.3%), the common carotid artery (CCA) in four patients (28.6%), and in one patient (7.1%), both the proximal and distal anastomotic sites of ICA. All patients were symptomatic, presenting with neck mass, dysphagia, transient ischemic attack, stroke, postoperative wound infection, or even rupture. The underlying etiology of ECAPAs was infection in five patients (35.7%), of which three had a history of prior neck surgery, trauma in four patients (28.6%), dissection in four patients (28.6%), giant cell arteritis in one patient (7.1%). The diameter of PAs ranged from 12.0 mm to 72.0 mm. Patient characteristics are summarized in the Table.

**Table:** Characteristics of patients in the study cohort

Pt	Sex	Age	Etiology	Site	Size, mm	Symptoms	Management
1	M	54	Mycotic	Left ICA	15.0 × 10.0	Painful neck mass, dysphagia, rupture	Resection, ligation of ICA
2	M	50	Disssection	Right ICA	15.0 × 20.0	Ischemic stroke	Resection, CEA, reimplantation of ICA + venous graft bypass
3	F	61	Disssection	Right CCA	15.0 × 30.0	Neck pain	Resection, prosthetic graft interposition
4	M	64	Disssection	Left ICA	15.0 × 15.0	TIA	Resection, CEA, reimplantation of ICA
5	M	64	Blunt trauma	Left ICA	72.0 × 55.0	Neck mass, dysphagia	Resection, ligation of ICA
6	M	55	Mycotic	Left ICA	40.0 × 35.0	Bleeding, p/о neck infection	Resection, ligation of CCA, ICA
7	M	46	Mycotic	Right CCA	20.0 × 10.0	Salivary fistula, p/о neck infection	Subclavian-carotid autogenous bypass
8	M	21	Penetrating trauma	Right ICA	30.0 × 30.0	Ischemic stroke	Resection, end-to-end anastomosis
9	M	57	Dissection	Right CCA	21.0 × 15.0	Ischemic stroke	Resection, prosthetic graft interposition
10	M	32	Blunt trauma	Left CCA	12.0 × 25.0	Neck mass	Resection, end-to-end anastomosis
11	F	57	Giant cell arteritis	Left ICA	12.0 × 6.0	Ischemic stroke	Resection, end-to-end anastomosis
12	M	49	Post-CEA, mycotic	Left ICA, proximal and distal anastomoses	16.0 × 27.0, 14.0 × 10.0	Neck mass, neck pain	Subclavian-carotid autogenous bypass, ligation of CCA, wound debridement
13	M	36	Gunshot shrapnel wound	Left ICA	15.0 × 17.0	Neck pain, dysphagia	Endovascular (stenting)
14	M	1 year 7 months	Mycotic	Right ICA	18.0 × 18.0	Peritonsillar abscess	Endovascular (stenting)

*CCA,* Common carotid artery; *CEA,* carotid endarterectomy; *F,* female; *ICA,* internal carotid artery; *M,* male; *TIA,* transient ischemic attack.

The choice of surgical technique was based on aneurysm location, size, and vessel condition, as well as the presence of infection. Classical surgical repair of carotid PAs included resection with primary anastomosis, carotid artery reimplantation, graft interposition or bypass, and carotid artery ligation.

Resection of PA with primary anastomosis was performed in three patients (21.4%), carotid artery reimplantation in two patients (14.3%), carotid artery ligation in three patients (21.4%), extra-anatomic autogenous subclavian-carotid bypass in two patients (14.3%), prosthetic graft interposition in two patients (14.3%), and endovascular treatment in two patients (14.3%).

**Fig 2:**
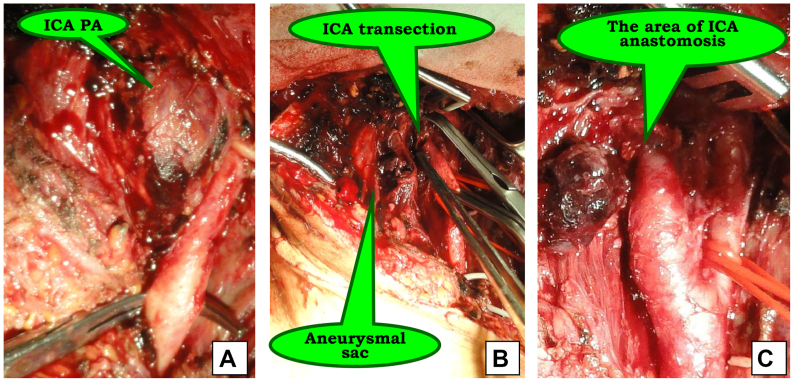
Penetrating neck trauma (stab wound). Intraoperative images. **(A)** Pseudoaneurysm (*PA*) of the internal carotid artery (*ICA*); **(B)** resection of PA; **(C)** completed view of reconstruction.

Adequate mobilization of the artery with healthy margins allowed the performance of end-to-end anastomosis (Fig 2).

**Fig 3:**
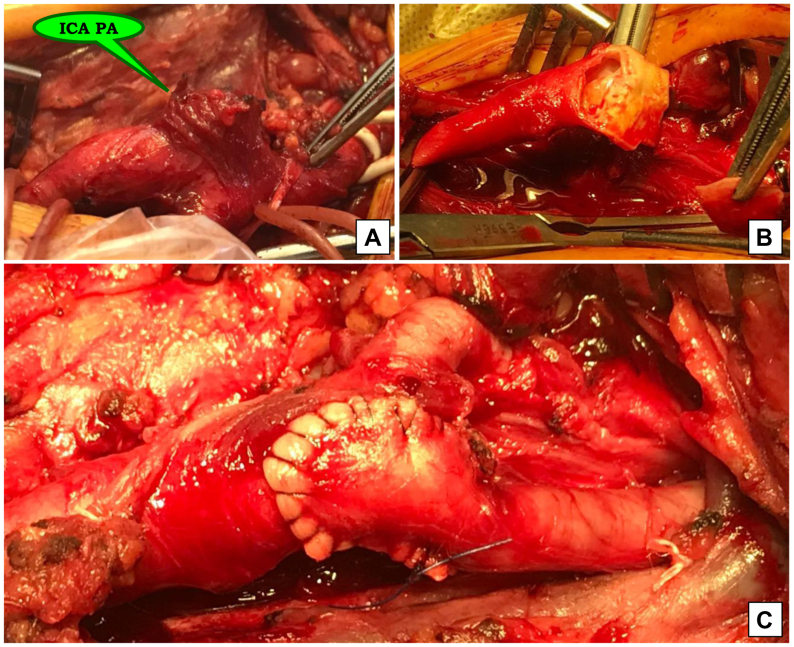
Intraoperative images. **(A)** Saccular pseudoaneurysm (*PA*) of the internal carotid artery (*ICA*); **(B)** resection of PA; **(C)** completed view of reconstruction.

In cases of small PAs with proximal involvement of the ICA, blood flow restoration was achieved by ICA reimplantation. Notably, giant cell arteritis was confirmed at histological study (Fig 3).

**Fig 4:**
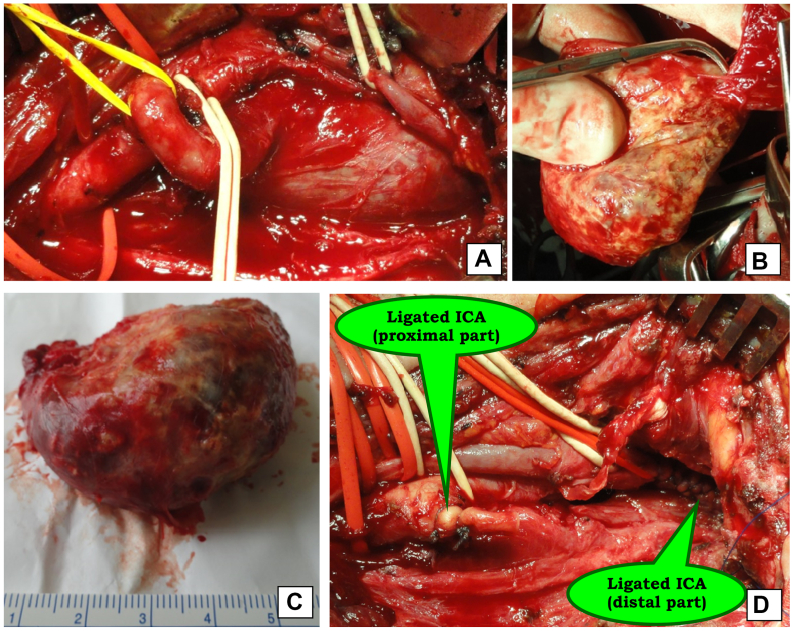
Intraoperative images. **(A)** Large pseudoaneurysm (PA) of the internal carotid artery (*ICA*); **(B)** mobilization of PA; **(C)** resected PA; **(D)** ligated ICA.

Ligation of the ICA was performed when reconstruction was not feasible due to large distal PA or emergency situations, such as arterial rupture (Fig 4).

Infectious ECAPAs were managed preferentially with autologous venous reconstruction, whereas noninfectious lesions, in the absence of clinical signs of infection and with unremarkable laboratory findings, were considered suitable for prosthetic graft placement. Microbiological cultures obtained from purulent material, soft tissues, or wound exudates served as the gold standard for confirming infection, with *Staphylococcus haemolyticus* and *Pseudomonas aeruginosa* being the most commonly identified organisms. Postoperatively, all patients with confirmed infection received empirical antibiotic therapy, followed by targeted therapy adapted according to culture results.

**Fig 5:**
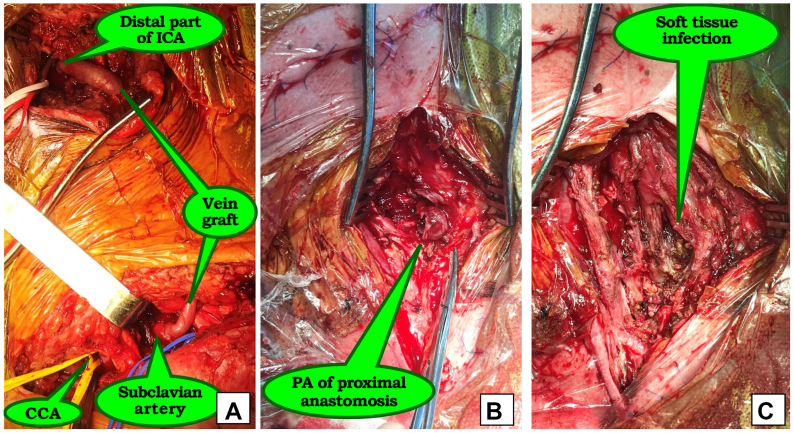
Intraoperative images. **(A)** Subclavian-carotid venous bypass; common carotid artery (*CCA*) was ligated to exclude pseudoaneurysms (*PAs*) from circulation; **(B)** via a separate surgical approach, PAs were resected, and the previous vein graft was removed; **(C)** intraoperative wound culture was obtained from infected soft tissues. *ICA*, Internal carotid artery.

One patient was admitted with an anastomotic PAs of the carotid vein graft following carotid endarterectomy (CEA) with bovine pericardial patch angioplasty for critical left ICA stenosis. The early postoperative period was complicated by patch rupture and a tense cervical hematoma. The patient subsequently underwent patch excision, external carotid artery ligation, and vein graft interposition from the CCA to the ICA. The decision was made to perform an extra-anatomic venous interpositional bypass from the subclavian artery to the ICA, ligation of the CCA, resection of the proximal and distal anastomotic PAs of the carotid vein graft, and wound debridement (Fig 5).

**Fig 6:**
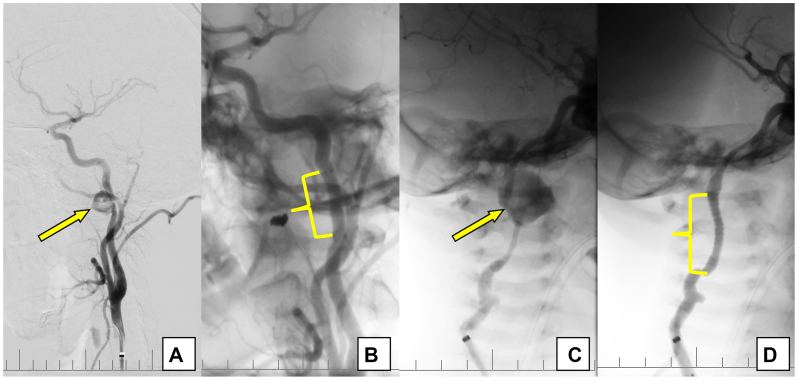
Angiograms of distal pseudoaneurysms (PAs) of the internal carotid artery (ICA), located near the skull base **(A** and **C)** and successful stent graft deployment **(B** and **D)**.

Endovascular stent graft deployment was performed in two patients with distal PAs of the ICA, located near the skull base (Fig 6).

Perioperative complications included: ischemic stroke in three patients (21.4%), cranial nerve injuries in two patients (14.3%), thrombosis of arterial reconstruction in two patients (14.3%), and wound infection in two patients (14.3%). Cranial nerve dysfunction was transient in all cases, with complete resolution within 30 days postoperatively. In one patient with thrombosis of ICA following CEA, thrombectomy with venous graft bypass from CCA to ICA was performed. Another patient with thrombosis of the subclavian-carotid vein graft had an asymptomatic course and did not require reoperation. Postoperative mortality rate was 7.1%: an ischemic stroke in a patient with a mycotic odontogenic PA complicated by diffuse purulent carotid arteritis.

Despite being the preferred approach in this cohort, open surgical repair was associated with a relatively high perioperative stroke rate of 21.4% (3 of 14 patients). Closer analysis revealed that all strokes occurred in high-risk anatomical or septic conditions where revascularization was either technically unfeasible or unsuccessful. In one case, ligation of the ICA was necessary due to active rupture of an odontogenic mycotic PA with purulent wall destruction. In another, distal ICA ectasia near the skull base with friable walls precluded safe reconstruction, necessitating ICA ligation. The third stroke occurred due to thrombosis after resection and reconstruction of a dissecting PA of the ICA associated with fibromuscular dysplasia. These observations suggest that perioperative stroke risk is higher when revascularization cannot be achieved, particularly in infected or structurally compromised vessels.

Long-term outcomes were assessed in 13 patients. The median follow-up was 21 months (range, 1–222 months). Overall, nine patients (69.2%) experienced no new neurological events, whereas one patient (7.7%) suffered a stroke during the follow-up period. Additionally, seven patients (53.8%) showed no carotid stenosis or occlusion. Two patients (15.4%) were lost to follow-up, and one patient (7.7%) died due to cancer progression.

## Discussion

Carotid interventions performed for aneurysms account for 0.2% to 5.0%.^7^^,^^8^ ECAPAs are even more rare with predominant involvement of ICA, because of its mobility and vulnerability for stretching.^9^ PA of ICA was first described by Liston as a rare complication of head and neck infection.^10^ From 1950 to 2012, there were 99 reported cases of ECAPAs.^11^ Since then, in current literary sources, there are enough publications, but mainly presented by small series of clinical observations or single clinical cases. Etiology of PA formation includes blunt trauma, infection, cancer, iatrogenic injury, prior neck surgery (CEA, angioplasty, and other operations), dissection, and radiation necrosis. Duplex ultrasonography, computed tomography angiography, and magnetic resonance angiography are the most common contemporary diagnostic modalities.^2^^,^^4^^,^^5^

Management of ECAPAs includes both surgical and endovascular methods, the use of which depends on the preferences of surgeons, as current guidelines do not provide clear recommendations.^3^^,^^12^, ^13^, ^14^, ^15^ Some authors recommend intervention for symptomatic ECAPAs and for asymptomatic ones exceeding 2.0 cm in diameter or with continuous growth.^16^ Resection of PA with arterial reconstruction is generally considered the gold standard of treatment, whereas endovascular technologies are reserved for cases with distal aneurysm location, previous neck surgery, prior radiotherapy, or in patients with high operative risk due to comorbidities. However, endovascular procedures carry risks of cerebral ischemia, do not relieve mass effect from large PAs, and may be technically challenging in tortuous carotid anatomy. The reported stroke rate after ligation of the ICA for ECAPAs varies widely across series, ranging historically from 11% to 32%. This variability depends on several factors, including aneurysm size and location, presence of infection or sepsis, and the adequacy of collateral circulation.^7^^,^^17^

Nevertheless, analyzing the existing literature, open repair has demonstrated high long-term patency. In a study by Ni et al, at 5-year follow-up, reintervention-free survival in the open repair group (90.9%) was higher than that in the endovascular repair group (69.2%).^3^ By the way, a synthetic graft demonstrated a 5-year primary patency rate of 88.9%, and a vein graft of 66.4%.^13^

In this clinical study, open surgical technique was the favored approach, with good long-term outcomes; endovascular treatment was reserved for selected patients with anatomically inaccessible distal PAs.

Thus, open surgery should be offered to young patients with ECAPAs, who have longer life expectancy, accessible surgical lesions, and acceptable surgical risk. A less-invasive endovascular approach may be prеfеrrеd in high-risk pаtiеnts with surgicаlly inаccеssible high PAs аnd hоstilе neck anatomy. However, due to the lack of randomized controlled trials, definitive conclusions regarding superiority of one method over the other remain challenging.^6^

This study has several limitations. The small sample size and retrospective design may limit the statistical power of the research. Additionally, the heterogeneity of patient etiologies and treatment methods complicates direct comparison of outcomes. Finally, the lack of a control group and relatively short follow-up period restrict the assessment of long-term efficacy and safety.

## Conclusions

ECAPA is a rare but potentially life-threatening condition that requires timely surgical intervention. The findings of this study suggest that reconstructive surgery can be an effective treatment, often resulting in favorable long-term outcomes. Further studies with larger cohorts and longer follow-up are needed to optimize management strategies and compare surgical vs endovascular approaches.

## Funding

None.

## Disclosures

None.
